# Heart Rate Asymmetry in Healthy Children

**DOI:** 10.3390/jcm12031194

**Published:** 2023-02-02

**Authors:** Dominika Zalas, Waldemar Bobkowski, Jarosław Piskorski, Przemysław Guzik

**Affiliations:** 1 Department of Pediatric Cardiology, Poznan University of Medical Sciences, 61-701 Poznań, Poland; 2 Institute of Physics, University of Zielona Gora, 65-516 Zielona Góra, Poland; 3 Department of Cardiology-Intensive Therapy, Poznan University of Medical Sciences, 61-701 Poznań, Poland

**Keywords:** heart rate asymmetry, children, heart rate variability, long-term ECG monitoring

## Abstract

Heart rate asymmetry (HRA) is a physiological phenomenon characterized by an unequal contribution of heart rate decelerations and accelerations to different heart rate variability (HRV) features. While HRA has been demonstrated in adults’ ECGs of different duration, a similar investigation in healthy children has not been conducted. This study investigated the variance- and number-based HRA features in 96 healthy children (50 girls and 46 boys, aged 3–18 years) using 24-h ECGs. Additionally, we studied sex differences in HRA. To quantify HRA, variance-based and relative contributions of heart rate decelerations to short-term (C1d), long-term (C2d), and total (CTd) HRV, and the number of all heartbeats (Nd) were computed. Heart rate decelerations contributed more to C1d, but less to C2d and CTd, and were less frequent than heart rate accelerations. Short-term HRA was better expressed in boys. The majority of children (93.7%) had short-term HRA, 88.5% had long-term HRA, 88.5% had total HRA, and 99.0% had more accelerations than decelerations. No sex differences were observed for the rate of various HRA features. Heart rate asymmetry is a common phenomenon in healthy children, as observed in 24-h ECGs. Our findings can be used as reference data for future clinical studies on HRA in children.

## 1. Introduction

Physiologically, the heart rate (HR) originates in the sinus node [1]. The frequency of its spontaneous depolarizations constantly fluctuates [2] due to various physical factors, such as temperature [3,4,5,6], and chemical factors, such as the concentration of electrolytes and hypoxia [7,8,9]. It is speculated that biological factors, such as sympathetic and parasympathetic activity, neurotransmitters, hormones, and cytokines, may also contribute to instantaneous HR [10,11,12,13].

The cardiac cycle duration is measured as the time between two consecutive R waves on an ECG (RR interval). Changes in HR can either be accelerations or decelerations. For RR intervals, these changes are either shortenings or prolongations, respectively. If the changes are of sinus rhythm origin, they are responsible for a physiological phenomenon known as heart rate variability (HRV) [14,15].

Different methods quantify HRV, e.g., variance-based methods (Poincaré plots, spectral analysis using Fast Fourier transformation, or Scargle–Lomb periodograms), entropy and detrended fluctuation analysis, or heart rate turbulence [16,17,18,19,20]. HRV has been chiefly used for two primary purposes: as an indirect measure of the autonomic control of the HR and as a predictor of clinical outcomes in various groups of patients.

Several HRV indices have been believed to be an indirect, non-invasive, and rough approximation of HR autonomic modulation, specifically sinus node activity. However, the 1996 Task Force document on HRV standards suggested that only short recordings of, for example, a 5-min duration in specific stationary conditions could be used for this purpose [21]. It has been debated and criticized, with some questioning whether HRV is a good proxy of the sympathovagal balance or vagal tone [22,23]. Recently, Marmerstein et al., based on an interesting animal study in rats, provided evidence that HRV parameters, which were assumed to reflect the vagal tone, are not correlated with vagal activity at all [24].

For longer ECGs, lasting hours to days, the original Task Force document on HRV has pointed out that HRV should not be employed to evaluate HR autonomic regulation [21]. HRV indices are measured globally for longer ECG recordings, such as 24-h, and have been documented only for risk prediction in various groups of patients. Some examples are adults who have survived myocardial infarction or have heart failure [25].

HRV has also been studied in various clinical and physiological studies. In adults, HRV indices change in different clinical conditions, such as acute coronary syndrome, heart failure, diabetes mellitus, hypertension, Parkinson’s disease [25,26,27], or physiological challenges—during exercise [28,29], orthostatic challenge [30,31], mental testing [32], and controlled breathing [33]. HRV has also been analyzed in healthy and ill children, for example, with congenital heart defects [34], diabetes mellitus [35,36], attention-deficit hyperactivity disorder (ADHD) [37], or after cardiac surgery [38]. Yet, HRV has never been systematically studied in healthy kids.

Physiologically, HRV changes with age [14,39,40,41,42,43,44,45,46,47,48,49,50]. Generally, many HRV indices increase during childhood and adolescence until approximately 20 years of age [46,51]. In adults, they gradually decline with age [52,53,54]. Some sex differences in HRV have also been demonstrated. For example, the standard deviation of the projection of the HRV Poincaré plot on the line of identity (SD2) was found to be higher in adult males [55,56]. However, the comparisons of HRV between girls and boys [39,40,41,42,43,44,45,46,47,48,51,57,58,59] show mixed results that sex differences may or may not be present in children.

As mentioned, HR accelerations and decelerations represent all RR changes creating HRV. It appears that HR accelerations and decelerations have an unequal input to short-term, long-term, and total HRV, and this physiological phenomenon is termed heart rate asymmetry (HRA) [18,19,30,60]. 

Our group showed in 2006 [18] and later [19,60,61,62,63] that HR decelerations contribute more to the short-term and less to the long-term and total HRV in ECG recordings of different lengths in adults [18,19,60,61,62,63]. Additionally, a phenomenon of HRA compensation exists—a larger input of HR decelerations to the short-term HRV compensates for a more considerable contribution of HR accelerations to the long-term HRV [61]. Most HRA investigations have been performed in shorter ECGs of 1- to 30-min duration and only a few on longer ECGs of 24-h duration [63,64,65]. 

It appears, however, that none of the HRA studies, including its compensation, were performed on the 24-h ECGs in healthy children. We assume that these two phenomena are present in healthy children. In the past, sex differences have been investigated in children only in HRV but never in HRA. 

We hypothesize that differences in the expression of HRA can be found in healthy children in long ECGs. Thus, we aimed to: (1) analyze the presence and expression of the short-, long-term, and total HRA in 24-h Holter ECGs collected from healthy children; (2) study the presence of HRA compensation in children; (3) compare the expression and rate of HRA and HRA compensation between girls and boys; and (4) explore the association between the age and HRA.

## 2. Materials and Methods

The Ethics Committee approved the study at Poznań University of Medical Sciences (file 549/10, appendix 351/19). Written informed consent was obtained from the parents or legal guardians of all children and participating children above seven years of age. Younger children who could not write gave oral consent in the presence of their parent or legal guardian.

### 2.1. Study Group

During 2017–2020, 1600 children were referred to our Out-patient Pediatric Cardiology Department for various reasons, such as heart murmur, impaired exercise tolerance, fainting, heart palpitations, and chest pain. These children underwent a thorough cardiac examination, which included medical history, physical examination, 12-lead ECG, and transthoracic echocardiography. 

For our study, 100 healthy subjects were selected, and the remaining 1500 children were ruled out based on the exclusion criteria: chronic disease, taking medications regularly, abnormalities on physical examination—except for the innocent heart murmur (confirmed on echocardiography), abnormal body mass index (BMI), laboratory measurements, blood pressure; any deviation from normal sinus rhythm on ECG, and abnormalities on echocardiography (such as valvular insufficiency or stenosis, septal defects, decreased myocardial contractility). Additionally, all children who performed professional sports or presented symptoms of infection four weeks prior to the visit were excluded as well. 

Although all children were referred to our Out-patient Pediatric Cardiology Department for potentially cardiovascular reasons, those reasons are also everyday problems in pediatric practice, not necessarily linked to an organic background. The most common example for referral is innocent heart murmurs which may be observed due to hyperkinetic circulation in healthy children, a poor physical condition may occur due to a sedentary lifestyle, syncope after prolonged standing or insufficient fluid consumption, heart palpitations, or chest pain of psychogenic background in stressful situations. 

The selected 100 children without any medical conditions underwent 24-h Holter ECG monitoring as the second step. Further exclusion criteria were a substantial number of supraventricular and ventricular extrasystoles (at least 100 over one hour), pairs or tachycardias, pathological bradycardia, non-sinus rhythm, atrioventricular conduction disturbances, or less than 90% of sinus rhythm on 24-h ECG Holter monitoring. The final number of included healthy children was 96.

### 2.2. 24-Hour ECG Holter Monitoring

Schiller’s Medilog^®^ Darwin 2 (Schiller, Switzerland) 3-channel ECG recorder with a sampling frequency of 1000 Hz was used for the 24-h ECG Holter monitoring. The ECG recordings were visually verified for beat misclassifications and manually corrected if needed. The minimum recording length was 18 h, and all recordings included night hours. The recordings were exported into text files of RR intervals for further calculations, and annotations about the beat type were added (sinus, supraventricular, ventricular, technical artifact).

### 2.3. Heart Rate Asymmetry Analysis

**Figure 1:**
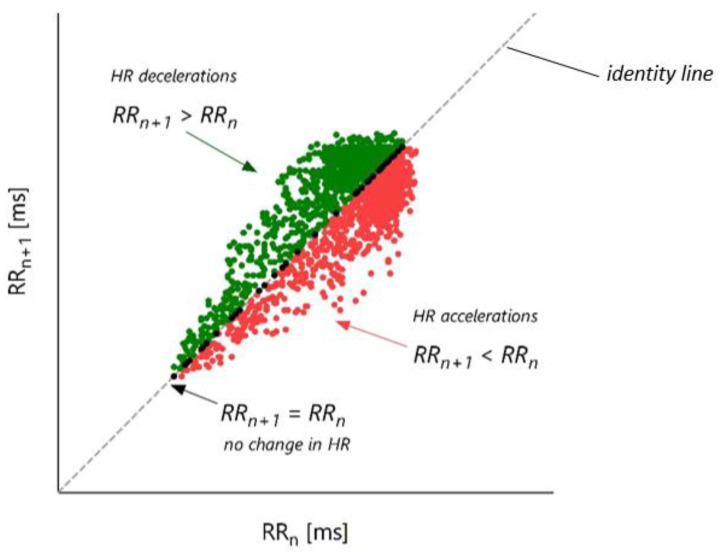
An example of the Poincaré plot of RR intervals. For clarity, instead of RR intervals from the 24-h ECG recording, which has, on average, 80,000–120,000 RR intervals, we present a set of RR intervals from a 30-min ECG. The whole plot is built by points described by pairs of two consecutive RR intervals, i.e., the current RR interval (RR_n_) and the following next RR interval (RR_n+1_). The Poincaré plot of RR intervals in the space (RR_n_, RR_n+1_) has many interesting physiological properties. Points described by RR intervals of the same duration (RR_n+1_ = RR_n_) (black dots) create the diagonal line (dashed grey line) termed the identity line. This line separates HR decelerations (green dots, RR_n+1_ > RR_n_) from HR accelerations (red dots, RR_n+1_ < RR_n_). Several variance-based descriptors describe the short-term, long-term, and total HRA [60,61,62,63,66].

For HRA analysis, we used the free-of-charge HRAExplorer software developed by Piskorski and Guzik [19], which is available at https://hraexplorer.com (accessed on 1 December 2022). All codes are available at https://github.com/jaropis/HRAexplorer (accessed on 1 December 2022). For more details, please refer to Piskorski and Guzik [19]. HRA analysis was based on the method of Poincaré plot of RR intervals (derived from ECG), as proposed by Guzik and Piskorski [19] (Figure 1), where the x-axis represents the current RR interval (RR_n_), and the y-axis represents the successive RR interval (RR_n+1_). The identity line represents points with the same current and successive RR intervals’ duration. Points above the identity line present HR decelerations, whereas points below it represent HR accelerations. SD1^2^ is the variance of the projection of points along the identity line, SD2^2^ is the variance of the projection of points perpendicular to the identity line, and SDNN^2^ is the variance of the projection of points on the RR_n_ axis.

Short-term HRV is measured by SD1, which represents the instantaneous beat-to-beat variability. It can be divided into parts related to HR decelerations and accelerations [66]:

$$\mathrm{SD}1^{2}=\;\mathrm{SD}1d^{2}+\mathrm{SD}1a^{2}$$

SD1^2^—variance of the dispersion of points in the Poincaré plot of RR intervals across the identity line.

SD1d^2^—part of SD1^2^ related to HR decelerations.

SD1a^2^—part of SD1^2^ related to HR accelerations.

For short-term HRA, the contribution of HR decelerations (C1d) is measured as follows:

$$C1d=\frac{\mathrm{SD}1d^{2}}{\mathrm{SD}1^{2}}$$

C1d—(also known as Guzik’s index [30]) contribution of HR decelerations to short-term HRV; SD1^2^ and SD1d^2^—see above. 

In short-term HRA contributions of HR decelerations and accelerations add up to 1:

C1d+C1a=1

C1a—contribution of HR accelerations to short-term HRV; C1d—see above.

Long-term HRV is measured by SD2, which represents the standard deviation of long-term continuous RR intervals. It can also be divided into parts related to HR decelerations and accelerations [66]:

$$\mathrm{SD}2^{2}=\;\mathrm{SD}2d^{2}+\mathrm{SD}2a^{2}$$

SD2^2^—variance of the dispersion of points in the Poincaré plots of RR intervals along the identity line.

SD2d^2^—part of SD2^2^ related to HR decelerations.

SD2a^2^—part of SD2^2^ related to HR accelerations.

For long-term HRA, the contribution of HR decelerations (C2d) is measured as follows:

$$C2d=\frac{\mathrm{SD}2d^{2}}{\mathrm{SD}2^{2}}$$

C2d—contribution of HR decelerations to long-term HRV; SD2^2^ and SD2d^2^—see above. 

By analogy, in long-term HRA, contributions of HR decelerations and accelerations add up to 1:

C2d+C2a=1

C2a—contribution of HR accelerations to long-term HRV; C2d—see above.

SDNN is the standard deviation of the duration of normal RR intervals and depends on short- and long-term HRV; it describes the total HRV [66]:

$$2{\mathrm{SDNN}}^{2}=\mathrm{SD}1^{2}+\;\mathrm{SD}2^{2}$$

SDNN^2^—variance of normal-to-normal RR intervals.

SDNNd^2^—part of SDNN^2^ related to HR decelerations.

SDNNa^2^—part of SDNN^2^ related to HR accelerations.

The total HRA is analyzed as follows:

$$\mathrm{CTd}=\frac{{\mathrm{SDNNd}}^{2}}{{\mathrm{SDNN}}^{2}}$$

CTd—contribution of HR decelerations to total HRV; SDNNd^2^, SDNN^2^—see above.

Analogically, in total HRA contributions of HR decelerations and accelerations sum up to 1:

CTd+CTa=1

CTa—contribution of HR accelerations to total HRV; CTd—see above.

For the analysis of the number of HR decelerations and accelerations, the parameter Nd was used. Its mathematical formula is as follows:

$$\mathrm{Nd}=\frac{\mathrm{nd}}{\mathrm{nd}+\mathrm{na}}$$

Nd—contribution of HR decelerations to the total number of RR intervals (also called Porta’s index).

nd—the absolute number of HR decelerations, na—the absolute number of HR accelerations.

The short-term HRA (HRA1) is present when C1d > 0.5, the long-term HRA (HRA2) exists when C2d < 0.5, and the total HRA (HRAT) is observed when CTd < 0.5. The HRA compensation (HRAcomp) phenomenon occurs when both C1d > 0.5 and C2d < 0.5 are present. Additionally, the HRA of the number of HR decelerations and accelerations (HRAN) is present when Nd < 0.5.

### 2.4. Statistical Analysis

The data distribution in most continuous parameters was not normal (Shapiro–Wilk test). Therefore, the nonparametric Mann–Whitney test was used, and the data were summarized as a median value, 25th, and 75th percentile (IQR—interquartile range). The comparison of HRA parameters between girls and boys was performed using the Mann–Whitney test. The occurrence rate of various types of HRA was checked using the binomial test, and the differences in the rates of HRA between girls and boys were examined with Fisher’s test. The correlation between age and continuous HRA variables was performed using the nonparametric Spearman correlation analysis. A *p*-value of <0.05 was considered statistically significant. All analyses were performed using MedCalc Statistical Software (MedCalc Software bv, Ostend, Belgium, https://www.medcalc.org; 2020, accessed on 1 December 2022).

## 3. Results

Ninety-six children aged 3 to 18 years participated in the study. The median age was 14 years, and 50 participants were girls. Table 1 summarizes the HRA results for all the studied children, and Table 2 presents the prevalence of different HRA forms and HRA compensation.

The prevalence of all types of heart rate asymmetry (HRA) exceeds 88% in all children, which is the highest for HRAN. HRA1 was present in more children than HRA2 and HRAT. All children with HRA2 also had HRAcomp.

The comparison between girls and boys (Table 1) revealed that the C1d parameter was significantly higher in boys, which suggests a stronger expression of short-term HRA in boys. No differences in the prevalence of either type of HRA or the HRA compensation phenomenon were observed between the sexes (Table 2).

Nonparametric Spearman rank correlation (Table 3) showed that age was significantly and positively correlated with SD2d, SD2a, SDNNd, and SDNNa. However, the correlations were weak to moderate, with rho between 0.31 for SDNNa and 0.42 for SD2d. No other HRA descriptors were found to change with age of the studied children.

## 4. Discussion

Our study demonstrates that heart rate asymmetry is present in 24-h ECG recordings of healthy children. This age group strongly expresses short-term, long-term, and total HRA and HRA compensation. Sex differences exist only in the expression of short-term HRA, which is slightly stronger in boys than in girls. However, the rate of HRA and its compensation are comparable for both sexes. The variance-based measures of long-term and total HRA were found to increase with the age of the children studied.

### 4.1. Asymmetry of Cardiovascular Time Series

HRA is not the only asymmetrical phenomenon demonstrated in the cardiovascular time series of adults. Using the beat-to-beat finger artery pressure waveforms, we demonstrated that the number and contribution of systolic blood pressure (SBP) increases to short-term blood pressure variability (BPV) were higher than of decreases in healthy adults [67]. The short-time variabilities of various atrial to His, His to ventricles, and the atrioventricular conductions are asymmetrical in one-minute intracardiac tracings [68]. Beat-to-beat 30-min values of stroke volume (SV), cardiac output (CO), and systemic vascular resistance (SVR), recorded non-invasively by cardiac impedance, also have asymmetric properties in healthy young people in a supine position. Increases in SV, CO, and SVR values contributed more than decreases in the short-term hemodynamic variabilities of these parameters [69].

Other phenomena related to the cardiovascular system also exhibit asymmetric features. A premature ventricular contraction triggers heart rate turbulence. It begins with short-lasting, usually 2–3 beats, HR accelerations and is followed by a series of HR decelerations for up to 15–20 beats [70,71,72,73].

Another example is the baroreflex regulating HR in response to blood pressure changes. The responses of the sinus node to changes in SBP are different, with baroreflex sensitivity being larger for SBP increases [74,75,76].

Recently, we have proposed a new method for studying spontaneous baroreflex function by focusing on its asymmetric properties [77,78]. Our findings suggest that short-term, long-term, total baroreflex sensitivity and baroreflex effectiveness are asymmetric in healthy individuals resting in a supine position. Increases in SBP had a larger contribution to the short-term baroreflex sensitivity but smaller to the long-term and total baroreflex sensitivity. These opposing effects of SBP increases on short-term and long-term baroreflex sensitivity demonstrate the existence of a compensatory phenomenon in the baroreflex function. 

### 4.2. Heart Rate Asymmetry in Children and Fetuses

Based on the studies of HRA in fetuses and neonates [79,80,81,82], it appears that HRA is already present prenatally and in the early postnatal period. A study of fetuses between 16 and 41 weeks of gestation showed the presence of short-term HRA in 95% of examined fetuses [79]. With the duration of pregnancy, no differences in C1d were observed [79]. The Nd index was smaller than 0.5 in 95% of analyzed fetuses and decreased with the duration of pregnancy. 

In another study of 44 fetuses during active labor (17 fetuses at 32–36 weeks of gestation and 27 at 38–40 weeks of gestation), where 10-min recordings of RR intervals were analyzed, it was proven that the short-term HRA was observed in both groups and its expression was significantly greater in the group of term fetuses [80]. 

Short-term HRA was also observed in healthy full-term infants, without prenatal and perinatal risk factors (at 72 h of chronological age), in ca. 25-min recordings of RR intervals. The values of C1d were different in resting conditions and under neonatal stress (heel stick blood drawing) [81]. 

In 70% of subjects in the neonatal group of a study by Czippelova et al. short-term HRA was present. It may suggest that HRA is a universal property even in the early stage of central nervous system maturation [82]. 

Few clinical studies have described HRA in children, but none have analyzed this phenomenon in 24-h ECG recordings of healthy children. In a study of 20 girls at an average age of 16.6 years with major depressive disorder (under resting conditions), it was found that their C1d was closer to 0.5, whereas the control group of 20 age-matched healthy girls had a higher C1d (>0.5) [83]. No significant differences between these groups in response to the orthostatic challenge were reported [83]. 

An age-matched study showed a reduction of C1d in 20 boys with ADHD at rest and in response to standing up, compared with 20 healthy boys [84]. Additionally, more reduced C1d was present in boys with ADHD after active standing [84]. In both studies, the short-term HRA was demonstrated in healthy children, and other HRA forms were not analyzed. However, the length of studied ECGs was short. 

In both studies, only segments of 300 RR intervals were used from smaller groups of only boys and in a narrower age range [83,84]. In contrast, we demonstrate the presence of all HRA forms in 24-h ECGs. Furthermore, our children group was larger and in a broader age range between 3 and 18 years, and included both boys and girls.

### 4.3. Heart Rate Asymmetry in Clinical Conditions

To date, HRA has been the subject of multiple studies in adults. It was analyzed in both healthy individuals during physiological challenges, and in patients with various diseases. 

Frank et al. analyzed 92 healthy students between 17 and 29 years of age, who were either training yoga or partaking in school sport once a week [28]. The short-term HRA (calculated from 20-min sleep recordings) increased significantly in the yoga group, from C1d = 0.48 to C1d = 0.51 [28]. 

ECGs of 5 min duration of adult females, who underwent tilt test, were compared between vasovagal and healthy groups [85]. The short-term HRA based on C1d was present in 31.6% of the healthy females in supine position and 63.2% in tilt, and in 56.3% of the vasovagal group in supine and 75% in tilt position. This suggests an interesting finding that the occurrence of HRA increases after tilting and it is more common in women who suffer from vasovagal syndrome [85]. 

Another study which examined orthostatic challenge was performed in 15 athletes aged 18–25 years and compared with 12 non-athlete individuals [86]. C1d was higher after upright standing only in athletes. Additionally, only in athletes did Nd increase in exercise as opposed to recovery [86]. 

Most studies have focused on short-term HRA (C1d or Guzik’s index) and the contribution of the number of decelerations to all changing beats (Porta’s index) without examining other HRA features. For example, HRA was significantly reduced in a study of 349 elderly patients (148 women) with heart failure and symptomatic aortic stenosis [87]. The reduction in HRA was correlated with the severity of heart failure according to the New York Heart Association (NYHA) classification [87]. Another smaller study analyzed 46 patients (9 women) with heart failure, and there were no significant differences in C1d between healthy subjects and patients with heart failure with the left-ventricular ejection fraction of 30% and NYHA functional class II and III [88]. 

Short-term HRA was reduced in adult patients with a history of diabetes (DM) type 1 for at least 25 years [65]. Based on 24-h ECG Holter monitoring, such patients had lower C1d than healthy volunteers [65]. If C1d equals or falls below 0.5, short-term HRA is lost—such findings were reported in patients with severe depression and DM type 2 [89]. 

Shi et al. for the first time discovered that HRA indices (C1d and Nd) were reduced in an oncologic disease—in 61 adult patients with gastric cancer [90]. Additionally, the decrease in Nd was positively correlated with the stage of cancer (as examined by the serum fibrinogen level) [90]. 

Treadmill training as a form of rehabilitation in post-stroke patients involves mental engagement [91]. Jelinek et al. found that HRA based on Nd parameter changes during such training, from values > 0.5, to values < 0.5., and they connected it with alterations in the ANS [91]. 

Similar disorders and clinical states in which HRA has been explored also affect children. However, in contrast to studies conducted in adults, there is a lack of reference data for HRA in healthy children. Our findings may serve as a database with typical values of HRA for future clinical and interventional studies, e.g., involving diet, physical training, relaxation techniques’ effects, and gaming’s impact.

### 4.4. Potential Mechanisms Involved in Heart Rate Asymmetry

The mechanisms of HRA remain not fully understood and are under continuous investigation, including our research group. Many potential mechanisms are potentially involved in HRA, such as those related to breathing rate and pattern, length of inspiration and expiration [33]. Other mechanisms are linked to various physiological reflexes, such as the baroreflex controlling the cardiovascular system, particularly the relationship between HR, blood pressure and vascular resistance [74]. 

HRA changes throughout the day and night [92], after an orthostatic challenge [93], and is a dynamic process [63]. Some studies suggest that HRA may originate from the asymmetrical behavior of other cardiovascular time series, such as SBP [67], hemodynamics [69], and atrioventricular conduction [68]. Karmakar et al. have also demonstrated that the short-term HRA is reduced after atropine infusion in healthy individuals, suggesting that vagal control of the HR might also be involved in this phenomenon [94]. 

In a study conducted on rats, Marmerstein et al. found no correlation between HRV indices and vagal activity [24]. Their research challenged the longstanding belief that HRV parameters can be used as a good approximation or indirect measure of autonomic control of the heart or sympathetic–parasympathetic balance. The evidence presented by Marmerstein et al. supports the positions of other prominent researchers in the field, such as D. Eckberg, G. Parati, and G. Mancia, who are cautious and critical about using HRV as a measure of sympathovagal balance [22,23]. Since there has been no demonstration that HRV depends on sympathovagal balance or vagal tone, we refrain from making such statements.

### 4.5. The Novelty of the Study

To date, a detailed exploration of HRA in 24-h ECGs of healthy children has not been conducted. We demonstrate the presence of HRA and HRA compensation in 24-h ECGs of healthy children and provide reference values for these phenomena in this age group. Moreover, while the expression of short-term HRA is stronger in boys than in girls, there were no differences in the prevalence of various forms of HRA and its compensation between the sexes. Finally, only variance-based long-term and total HRA significantly alter with the age of the healthy children. 

### 4.6. Limitations of the Study

In our study, we did not consider the pubertal development of our children in order to avoid further subdivisions of our data, reduction of statistical power, and drawing unjustified conclusions. No studied children were younger than three years, as collecting reliable 24-h ECGs is challenging in this age group. We have studied HRA only in children of the Caucasian race because most of the Polish population are ethnic Poles with typical features for Slavic people. Consequently, our study group was racially homogenous, and any conclusion extrapolated to other ethnic groups should be considered uncertain. Additionally, this study is observational, and its clinical significance is unknown.

## 5. Conclusions

We show that HRA and its compensation are present in healthy children in 24-h ECG recordings. The various properties of HRA are found in most (at least 88%) healthy children aged 3–18 years. Differences between girls and boys were found only in short-term HRA (which is better expressed in boys), and no differences in the prevalence of HRA were found between the sexes. The long-term and total HRA develop with age. 

Further research is needed to fully understand HRA’s potential mechanisms and clinical significance in children.

## Figures and Tables

**Table 1:** HRA parameters in the whole group (n = 96) and compared between girls (n = 50) and boys (n = 46) (Mann–Whitney test; * *p* < 0.05).

Parameter	All Children		Girls		Boys		*p*-Value
	Median	IQR	Median	IQR	Median	IQR	
SD1d (ms)	34.25	21.33–43.97	32.04	20.01–43.40	36.13	24.69–44.64	0.36
SD1a (ms)	29.21	19.80–37.88	28.83	19.13–37.49	30.09	21.19–40.08	0.50
SD2d (ms)	153.07	117.68–177.64	153.59	117.65–177.79	148.72	117.71–177.48	0.97
SD2a (ms)	158.35	126.79–191.71	160.02	127.44–193.25	156.22	124.79–191.50	0.82
SDNNd (ms)	111.49	88.63–129.36	112.27	90.15–128.58	109.89	86.43–129.72	0.96
SDNNa (ms)	114.96	91.77–137.54	114.98	92.71–137.61	113.61	91.32–137.47	0.83
C1d	0.56	0.53–0.58	0.55	0.51–0.57	0.57	0.53–0.58	0.03 *
C2d	0.48	0.46–0.49	0.48	0.46–0.49	0.47	0.46–0.49	0.22
CTd	0.48	0.47–0.49	0.48	0.47–0.49	0.47	0.46–0.49	0.24
Nd	0.42	0.39–0.45	0.42	0.39–0.44	0.43	0.40–0.45	0.35

C1d—contribution of HR decelerations to the short-term HRV; C2d—contribution of HR decelerations to the long-term HRV; CTd—contribution of HR decelerations to the total HRV; IQR—interquartile range; Nd—contribution of the number of HR decelerations to the total number of RR intervals; SD1a—part of SD1^2^ related to HR accelerations (square root); SD1d—part of SD1^2^ related to HR decelerations (square root); SD2a—part of SD2^2^ related to HR accelerations (square root); SD2d—part of SD2^2^ related to HR decelerations (square root); SDNNa—part of SDNN^2^ related to HR accelerations (square root); SDNNd—part of SDNN^2^ related to HR decelerations (square root).

**Table 2:** Prevalence of different types of HRA in the whole group (n = 96) and compared between girls (n = 50) and boys (n = 46) (Fisher’s exact test).

Prevalence					
	All Children (%)	*p*-Value	Girls (%)	Boys (%)	*p*-Value
HRA1	90 (93.7)	<0.001	45 (90.0)	45 (97.8)	0.12
HRA2	85 (88.5)	<0.001	44 (88.0)	41 (89.1)	0.86
HRAT	85 (88.5)	<0.001	44 (88.0)	41 (89.1)	0.86
HRAN	95 (99.0)	<0.001	49 (98.0)	46 (89.1)	0.34
HRAcomp	85 (88.5)	<0.001	44 (88.0)	41 (89.1)	0.86

HRA1—short-term HRA; HRA2—long-term HRA; HRAcomp—HRA compensation; HRAN—HRA of the number of HR decelerations and accelerations; HRAT—total HRA.

**Table 3:** The correlation between age and continuous HRA descriptors (n = 96) (Spearman’s rank correlation coefficient).

Parameter	rho	SE of rho	*p*-Value
SD1d	−0.04	0.1031	0.7148
SD1a	−0.05	0.1030	0.6592
SD2d	0.42	0.0938	<0.0001
SD2a	0.34	0.0970	0.0007
SDNNd	0.36	0.0962	0.0003
SDNNa	0.31	0.0979	0.0018
C1d	0.01	0.10	0.9729
C2d	0.05	0.10	0.5967
CTd	0.04	0.10	0.7179
Nd	0.03	0.1031	0.7353

C1d—contribution of HR decelerations to the short-term HRV; C2d—contribution of HR decelerations to the long-term HRV; CTd—contribution of HR decelerations to the total HRV; Nd—contribution of the number of HR decelerations to the total number of RR intervals; SD1a—part of SD1^2^ related to HR accelerations (square root); SD1d—part of SD1^2^ related to HR decelerations (square root); SD2a—part of SD2^2^ related to HR accelerations (square root); SD2d—part of SD2^2^ related to HR decelerations (square root); SDNNa—part of SDNN^2^ related to HR accelerations (square root); SDNNd—part of SDNN^2^ related to HR decelerations (square root); SE—standard error.

## Data Availability

The data presented in this study are available on request from the corresponding author.
